# Functional Neurological Symptom Disorder: A Diagnostic Algorithm

**DOI:** 10.1155/2019/3154849

**Published:** 2019-07-25

**Authors:** Eugénie Girouard, Isabelle Savoie, Ludivine Chamard Witkowski

**Affiliations:** ^1^Neurology, Dr. Georges-L.-Dumont University Hospital Centre, Centre de Formation Médicale du Nouveau-Brunswick, Moncton, NB, Canada; ^2^Nuclear Medicine, Dr. Georges-L.-Dumont University Hospital Centre, Moncton, NB, Canada

## Abstract

Functional neurological symptom disorder (FNSD) is a neuropsychiatric disorder characterized by the presence of neurological symptoms in the absence of any neurological abnormality that can be linked to a known pathology. Few studies have taken interest in this subject probably because of the heterogeneity of results. It is most often a diagnosis of exclusion which often means that patients undergo many tests and find themselves erring for a diagnosis with very little satisfaction of the outcomes. A reliable imagery pattern would therefore provide some relief and confirmation for both patients and clinicians. It could also facilitate acceptation of the diagnosis and reduce the societal cost associated with FNSD for the patient. The aim of this present study was to describe a clinicoradiological correspondence algorithm of FNSD using the PET scan and SPECT scan (PoSPs) and grant the clinician with a reliable tool to facilitate the diagnosis of FNSD. A systematic review according to the 2009 PRISMA criteria statement was used to guide the review. Our study included 3 of our own consenting patients who met the Diagnostic and Statistical Manual of Mental Disorders 5^th^ edition criteria as well as 25 other patients from 7 different studies. Our results showed a hypoactivation with poor clinicoradiological correspondence and poor stability in time. This hypoactivation was mostly in the frontal lobe, which could explain some behavioral alterations. These findings oppose the ones found in organic pathologies and therefore should orient towards FNSD. In the light of these findings, we recommend the clinicians to perform two PoSPs, searching for clinicoradiological lack of correspondence and time stability using our algorithm.

## 1. Introduction

Functional neurological symptom disorder (FNSD), also known as conversion disorder, is defined by the Diagnostic and Statistical Manual of Mental Disorders 5^th^ edition as abnormal central nervous system functioning of presumed psychogenic etiology [1]. The manifestations can include, but are not limited to, weakness or paralysis; abnormal movement such as tremor, dystonia, myoclonus, and gait disturbances; dysphagia; difficulties speaking such as dysphonia or slurred speech; attacks or seizures; anesthesia or sensory loss; and hearing, visual, and olfactive anomalies. It can further be classified in an acute episode if below six months or persistent episode if above six months and with or without psychological stressor prior to the episode. Emotional stressors could have been a contributing causal factor to the development of FNSD with abnormal movement characteristics [2]. It would act by inhibiting the descending motor pathways through the amygdala and orbitofrontal cortex. The prevalence of FNSD is currently unknown, but percentage of secondary assessment in neurology clinics is estimated to be 5%. Although the incidence is estimated to be 2-5/100,000 cases per year, this disorder seems to be more common than previously thought [1]. For example, 20-25% of patients admitted in neurology wards are estimated to have FNSD symptoms [3].

Few studies have taken interest in this subject probably because of the heterogeneity of results. Previous research has shown that FNSD could be explained by an abnormal limbic regulation with increased amygdala activity and engagement of prefrontal cortex, periaqueductal gray area, and basal ganglia [4]. These would result in abnormal ventromedial prefrontal (vmPFC) cortex activity. More recently, a general model to explain FNSD pathophysiology has been proposed by Conejero et al. [5]. According to their proposed model, FNSD would be linked to functional and structural abnormalities. These could be grouped into five distinct systems. The self-focused attention and action monitoring, both linked to the vmPFC and the precuneus, showed an abnormal activation on the PET scan and SPECT scan (PoSPs). The salience network, linked to the amygdala, the insula, and the anterior cingulate cortex (ACC), showed an abnormal activation which could lead to altered selection of motor patterns in the supplementary motor area and the dorsolateral prefrontal cortex (DLPFC). The self-agency network, linked to the DLPFC, the temporoparietal junction (TPJ), and the angular gyrus, showed hypoactivation on imagery. The memory suppression system, linked to the DLPFC also showed hypoactivation on imagery. Although this model provides important cues to the elucidation of FNSD pathophysiology, it needs further confirmation by prospective studies to be validated.

The diagnosis of FNSD is often difficult and remains a diagnosis of elimination. Few clinical signs have been demonstrated to have decent clinical specificity and sensitivity. Physical exam findings include motor signs (Hoover's sign, abductor's sign, abductor finger sign, spinal injury test, collapsing or giveaway weakness, cocontraction, and motor inconsistency), sensory signs (midline splitting, splitting of vibration, nonanatomical sensory loss, inconsistency or changing pattern sensory loss, and systemic failure), and gait signs (dragging monoplegic gait and chair test) [6]. Other clues can be used to aid the diagnosis, but it should be noted that these are not specific. They include history of similar symptoms; temporospatial link with symptomatology and experienced stressor or trauma; association with dissociative symptoms such as depersonalization, derealisation, or dissociative amnesia [1]; female sex; onset in late childhood or early adulthood; and La belle indifference [3].

Approximately 83% of patients will see an improvement in their symptoms in the following 4-6 years after the diagnosis [3]. Short duration of symptoms and acceptance of the diagnosis are important positive prognosis factors [1]. In spite of these signs, the diagnosis of FNSD remains a difficult diagnosis of elimination, which relies on a set of arguments. A reliable imagery pattern would therefore provide some relief and confirmation for both patients and clinicians. It could also facilitate acceptation of the diagnosis and reduce the societal cost associated with FNSD for the patient.

Single Photon Emission Computed Tomography (SPECT) is a 3D imaging technique using gamma ray tracers. SPECT scans produce a gross image that can be very useful in diagnosing and following the progression of various diseases. It has recently been suggested that SPECT imagery could aid with the diagnosis of Parkinson's disease by distinguishing it from other movement disorders. Position Emission Tomography (PET) is also a 3D imaging technique, but instead of using gamma ray tracers, it requires radiotracers. PET scans produce an image of higher quality and is commonly used to detect cancers and monitor their progression. The Food and Drug Administration has also just recently approved PET scan utilization to differentiate Alzheimer's disease from other forms of dementia [7]. The functional mechanisms of FNSD have previously been studied with both SPECT and PET scans [5], but we believe we are one of the first to attempt FNSD diagnosis using a special algorithm with these imagery techniques.

## 2. Materials and Methods

### 2.1. Literature Review

Our study included 3 of our own consenting patients who met the DSM-5 criteria as well as 25 other patients from 7 different studies.

**Figure 1:**
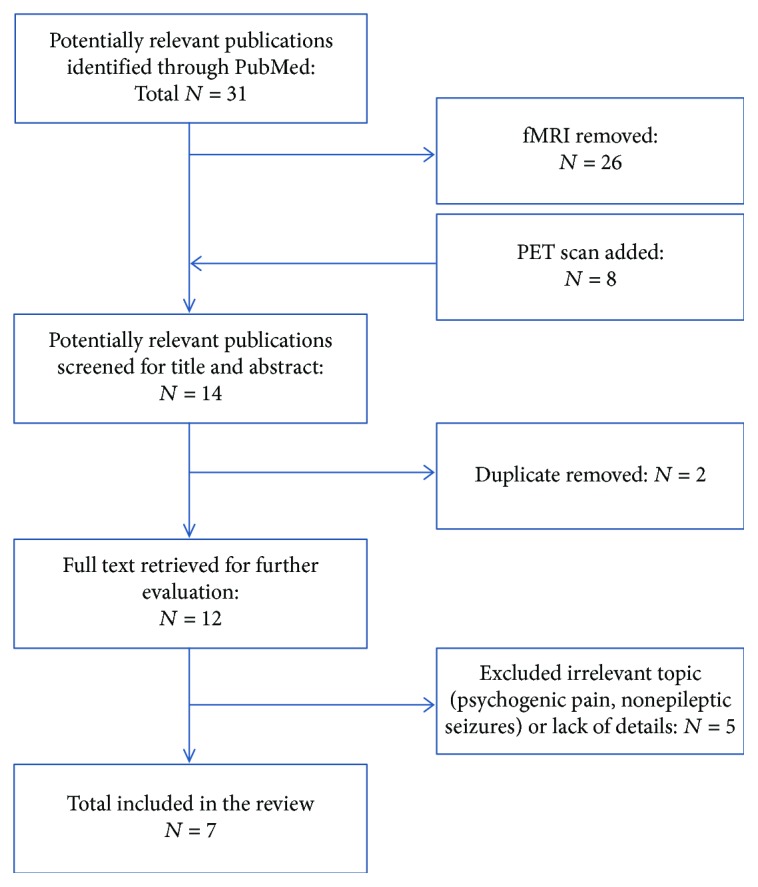
Study flow chart (*N*: number of scientific publications).

A systematic review according to the 2009 PRISMA criteria [8] statement was used to guide the review. Searches were conducted on PubMed for potentially relevant studies without date restrictions through October 2018. We only screened the English and French publications. The keywords applied for this search were [((somatoform) OR (conversion) OR (functional)) AND ((99mTc HMPAO) OR (SPECT) OR (perfusion))] as well as [((somatoform) OR (conversion) OR (functional)) AND (PET)]. Titles and abstracts were identified by two independent reviewers. We included original study concerning PET scan or SPECT scan and used exclusion criteria to assure homogeneity and specificity as follows: (1) studies concerning psychogenic pain, (2) studies concerning functional MRI, (3) studies concerning non epileptic seizures, (4) studies providing not enough clinical details, and (5) patients with comorbidities such as organic pathologies. Overall, a total of 43 publications were found; 26 were eliminated by the title and 7 by complete reading (see Figure 1). Then, a total of 7 publications were included in this review. We collected the clinical and radiological information for every patient (our 3 patients and 25 from the 7 publications) such as sex, age, hand dominance, predominant symptom(s), duration of disease, resolution or relapses, comorbidities, and PoSP results.

Each patient was classified into the appropriate category according to the DSM-5 criteria. The categories were “with weakness or paralysis,” “with abnormal movement,” “with swallowing symptoms,” “with speech symptoms,” “with anesthesia or sensory loss,” “with special sensory symptoms,” and “with mixed symptoms.”

### 2.2. SPECT Scans

Each of our consenting patients went through a first series of SPECT scans with injection of 99mTc-HMPAO to measure cerebral perfusion. Scans were repeated in order to establish the evolution of each patient. The SPECT scans were preferred to fMRI or PET scan due to high cost and limited availability of these techniques.

Due to the limited specificity of SPECT scans, a simplified anatomical-functional correspondence was used in an attempt to correlate the SPECT scan results with the patient's syndrome. For each patient, we took the most predominant symptom. Each specifier recognized by the DSM-5 was matched to an area of the brain as follows: weakness or paralysis with the frontal lobe contralateral to the deficit, abnormal movement with basal ganglia, swallowing symptoms with the brain stem, speech symptoms with the left parietal or frontal lobe, anesthesia or sensitive loss with the parietal lobe contralateral to the deficit, and mixed symptoms with their respective anatomical-functional associations [9]. Our algorithm is presented further in this article. Positive symptoms (abnormal movements, various sensations) were thought of as showing hyperactivation on imagery whereas negative symptoms (motor deficit, hypoesthesia, and aphasia) were thought of as showing hypoactivation on imagery. We then linked each patient's symptomatology to the expected area according to our algorithm and verified if they matched with PET or SPECT results. When 2 PoSPs were available, we evaluated stability in time between 2 examinations. This algorithm was created according to organic pathology correspondence. In the case of our FNSD patients, we expected that the correspondence, as gross as it may be, would not be verified. We also expected a limited stability in time.

## 3. Results

We included 3 of our patients and 25 patients from the literature review. Of the 29 patients we studied, 44% of them were women. The mean age was 43.41 years. The mean duration of the symptomatology was 21.23 months.

The PoSP results were abnormal for 85.7% of patients and always showed hypoactivation despite 12% of patients having positive symptoms. As described in Table 1, the hypoactivation was in one (62.5%), two (17%), or more than three (21%) regions. The frontal lobe (46%), parietal lobe (38%), temporal lobe (29%), basal ganglia (29%), brain stem (17%), and occipital lobe (4%) were affected.

The percentage of patients who experienced resolution of symptoms was 53.8%.

Out of the 11 patients with repeated scans, the percentage of patients with stable findings on imagery was 27%. The percentage of patients matching our anatomical-functional correspondence was 52.9%. The percentage of patients whose symptom was shown in the appropriate hemisphere was 76.5%.

### 3.1. Our 3 Patients' Clinical Description

Patient 1 was a right-handed woman of 72 years old with a known history of anxiety. She experienced tremors of the tongue and both arms. She complained of fatigue, memory impairment, and important tremors, which were more important in her right side. When distracted, her physical exam appeared to be normal. Her symptoms persisted for a total of 180 months, and she has experienced no remission.

**Figure 2:**
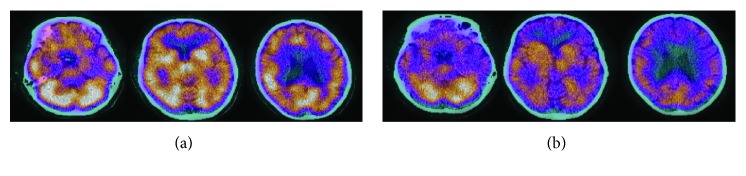
Colour scale brain perfusion SPECT analysis with 99mTx-HMPAO fused with CT scan for patient 1: (a) December 2017; (b) July 2018.

As shown in Figure 2, the first scan revealed moderate bilateral hypoperfusion in the frontal, temporal, and parietal lobes. This hypoperfusion was more pronounced on the left side of the brain. On repeated scan, findings were similar but with a much more severe global hypoperfusion of the brain.

Patient 2 was a right-handed man of 47 years old with a known history of depression. He experienced a sensorimotor deficit on the left side of his body. He complained of fatigue, memory impairment, diffuse pain paraesthesia, and muscle spasms. His neurological exam appeared normal. His symptoms persisted for a total of 24 months, and he has experienced no remission.

**Figure 3:**
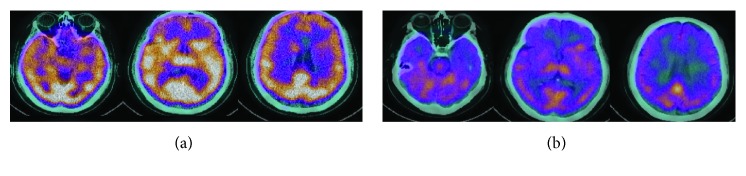
Colour scale brain perfusion SPECT analysis with 99mTx-HMPAO fused with CT scan for patient 2: (a) April 2018; (b) August 2018.

As shown in Figure 3, the first scan revealed moderate bilateral hypoperfusion of the frontal, temporal, and parietal lobes. These changes were more pronounced in the frontal lobes. On repeated scans, the brain shows severe generalized hypoperfusion.

Patient 3 was a right-handed man of 45 years old. He experienced a motor deficit in his left arm. He also complained of memory impairment, abulia, sexual dysfunction, and difficulty walking. When distracted, his physical exam was normal. His symptoms persisted for a total of 12 months, and he has experienced a near-total remission.

**Figure 4:**
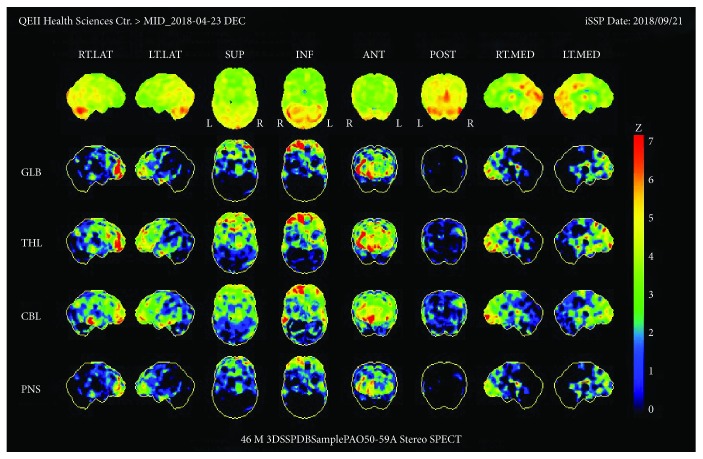
Colour scale brain perfusion SPECT analysis with 99mTx-HMPAO for patient 3.

As shown in Figure 4, the scan revealed mild hypoperfusion in the left medial and inferior temporal lobes as well as decreased parietal perfusion slightly more pronounced on the left side.

## 4. Discussion

### 4.1. Pet and SPECT Scan (PoSP) Results

As described above, PoSP results were abnormal for the majority of patients. Abnormal imagery each showed hypoactivation regardless of positive or negative symptoms. This opposes organic pathologies in which positive symptoms would result in hyperactivation and negative symptoms in hypoactivation [9]. Hypoactivation was mainly restricted to one region, and that region was most often the frontal lobe and the parietal lobe. These findings are congruent to the theory of Conejero in which the hypoactivation of self-agency network could explain pathophysiology of FNSD. The self-agency network, linked to the DLPFC, the temporoparietal junction (TPJ), and the angular gyrus, plays a role in the judgement of action by comparison of internal modes of action and sensory feedback [5]. They therefore can be linked to inhibition of motor selection patterns.

Furthermore, each hypoactivated region had very little correspondence to the expected clinicoradiological region. On the other hand, organic pathologies, such as stroke or epilepsy, have a good correspondence between symptomatology and imagery findings [9]. These were not the case for the majority of FNSD patients. However, the majority of the patient's symptoms were linked to the appropriate hemisphere which is also the case for organic pathologies. Dissociation reported and sought in a clinical exam (Hoover's sign, etc.) is also found in PoSP results [5].

Out of the 11 patients with repeated scans, the majority of them did not have stable imagery findings. This opposes with chronic organic pathologies in which a brain anomaly will generally persist on scans [6]. Some, but not all, patients have experienced a resolution of symptoms. Generally, patients should see some improvement over the course of 4-6 years [3], which also does not correlate with organic pathology. An important factor towards improvement is the acceptance of the diagnosis [1]. This is why we recommend the use of our clinical algorithm to aid not only the clinician in their diagnosis but also the patient in the acceptation of their diagnosis. Furthermore, we recommend that the clinician show the SPECT results to the patient. This will allow for a patient-centred approach where the patient will feel empowered and understood in their suffering. It will also help them find comfort in the fact that they are not feigners but rather they suffer from a well-known, diagnosed disease. The goal is to help the patient accept the diagnosis and patient care as well as avoidance of useless further exams and consultations. Furthermore, identifying a specific pattern of central nervous system dysfunction would help to reduce stigmatisation of these patients.

### 4.2. Clinical Algorithm

**Figure 5:**
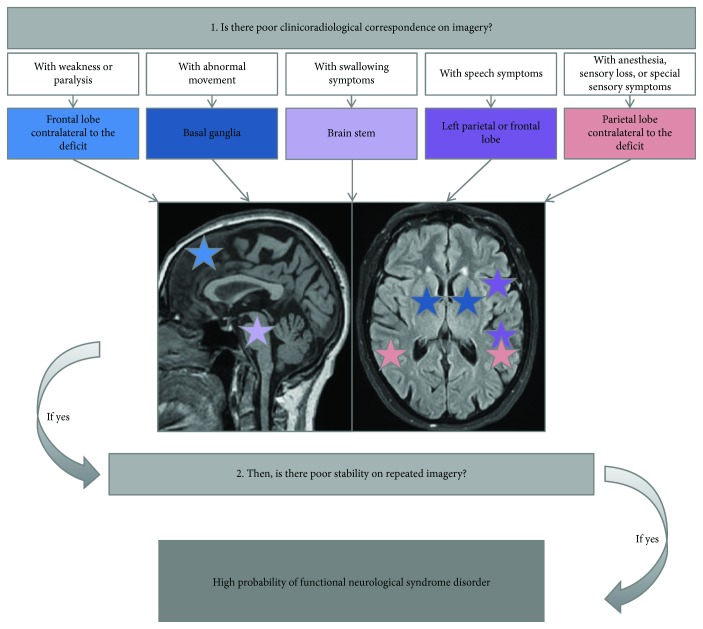
Diagnostic algorithm for the clinician to use in suspicion of FNSD using clinicoradiological correspondence on imagery according to DSM-5 specifiers. Each colour-coded star matched to an expected brain area of hypoperfusion (for negative symptoms) or hyperperfusion (for positive symptoms) according to patient symptomatology for organic pathologies.

The clinician should consider FNSD when the patient meets the DSM-5 criteria, and neurological symptoms cannot be explained by clinical findings demonstrated by a physical exam or imagery. Incongruence between symptoms and the affected brain region as described in Figure 5 and inconsistency of PoSP findings through the course and evolution of the disease should warrant FNSD. Although less specific, hypoactivation of the frontal lobe should also raise suspicion of FNSD.

### 4.3. Limitations

Since very little interest has previously been manifested in FNSD research, results can show important heterogeneity. Patients are also issued from various publications, which means there is significant variation in methodology and certain data could be missing. Therefore, data was not suitable for accurate statistical analysis. As for our own patients, all data was collected in retrospective as opposed to prospective studies which could lead to recall bias. Most patients presented with a wide range of symptoms. For concerns of clarity, we had to target one or two predominant symptoms, which could lead to missing information. Another important limitation to FNSD research is that most patients suffering from FNSD also suffer from psychiatric comorbidities. These conditions can also show hypoactivation, poor clinicoradiological stability, and poor stability in time. Further studies with psychiatric controls would be required to properly distinguish the neurobiological difference between FNSD and psychiatric comorbidities. The use the SPECT scan provides gross imagery with lesser specificity, but due to its superior availability and lower cost, it was chosen instead of the PET scan. The size of our patient group is also limited. The strength of our algorithm increased with the less specific anatomic correspondence region. This is why gross correspondence regions were attributed to symptomatology. Our algorithm therefore has potential to become a useful diagnostic tool, but due to facts stated above, it remains a relief to the patients and the physician. As more research is done to elucidate FNSD, further specificity should arise. Prospective studies with a psychiatric patient control group should be expected as the pathophysiology of FNSD is further elucidated and more advanced imageries are developed.

## 5. Conclusions

PoSPs usually showed hypoactivation in FNSD with a poor clinicoradiological correspondence and poor stability in time, in contrast to organic pathologies. The implication of the frontal lobe could also demonstrate a behavioral or conduct disorder. We recommend the clinicians to assess for DSM-5 criteria, perform a detailed physical examination, and perform two PoSPs, searching for clinicoradiological correspondence and time stability using our algorithm. Use of our algorithm should provide some relief and confirmation for both patients and clinicians. It could also facilitate acceptation of the diagnosis, improve the prognosis, reduce stigmatisation, and reduce societal cost associated with FNSD for the patient. A prospective study should be expected.

## Figures and Tables

**Table 1:** Patient's symptoms and PoSP results.

Source	Symptom	DSM-5	Expected results	PoSP results	Repeated scan
Our patient 1	Tremor (tongue and both arms)	Abnormal movement	Hyperperfusion basal ganglia	Hypoperfusion fronto-temporo-parietal bilateral	No stability

Our patient 2	Left sensorimotor deficit	Mixed (motor+sensory loss)	Hypoperfusion right frontoparietal lobe	Hypoperfusion fronto-temporo -parietal bilateral	No stability

Our patient 3	Motor deficit (left arm)	Weakness or paralysis	Hypoperfusion right frontal lobe	Hypoperfusion temporoparietal left	NA

Tiihonen et al. [10]	Left sensorimotor deficit	Mixed (motor+sensory loss)	Hypoperfusion right frontoparietal lobe	Hypoperfusion frontoparietal right	No stability

Yazici and Kostakoglu [11]	Imbalance, expression aphasia	Speech	Hypoperfusion left parietal lobe	Hypoperfusion parietal left	NA

Gürses et al. [12]	Imbalance, tremor of 4 limbs	Abnormal movement	Hyperperfusion left parietotemporal lobe	Hypoperfusion parietotemporal left	No stability

Vuilleumier et al. [13]	Left arm sensory deficit	Anesthesia or sensory loss	Hypoperfusion right parietal lobe	Hypoperfusion basal ganglia right	No stability
	Left sensorimotor deficit	Mixed (motor+sensory loss)	Hypoperfusion right frontoparietal lobe	Hypoperfusion basal ganglia right	No stability
	Right motor deficit	Weakness or paralysis	Hypoperfusion left frontal lobe	Hypoperfusion basal ganglia left	No stability
	Right leg sensorimotor deficit	Mixed (motor+sensory loss)	Hypoperfusion left frontoparietal lobe	Hypoperfusion basal ganglia left	No stability
	Left motor deficit, right sensory deficit	Mixed (motor+sensory loss)	Hypoperfusion right frontal lobe, left parietal lobe	Hypoperfusion basal ganglia bilateral	Stable in time
	Left motor deficit	Weakness or paralysis	Hypoperfusion right frontal lobe	Hypoperfusion basal ganglia right	Stable in time
	Right sensory deficit, motor deficit of 4 limbs	Mixed (motor+sensory loss)	Hypoperfusion left parietal lobe, bilateral frontal lobes	Hypoperfusion basal ganglia right	Stable in time

Garcia-Campayo et al. [14]	Left-sided manifestations^∗^	NA	NA	Normal	NA
	Left-sided manifestations^∗^	NA	NA	Hypoperfusion brain stem right	NA
	Right-sided manifestations^∗^	NA	NA	Hypoperfusion frontal left, brain stem right	NA
	Bilateral manifestations^∗^	NA	NA	Hypoperfusion frontal	NA
	Bilateral manifestations^∗^	NA	NA	Normal	NA
	Left-sided manifestations^∗^	NA	NA	Hypoperfusion brain stem right	NA
	Bilateral manifestations^∗^	NA	NA	Normal	NA
	Left-sided manifestations^∗^	NA	NA	Hypoperfusion frontotemporo parietooccipital right	NA
	Left-sided manifestations^∗^	NA	NA	Normal	NA
	Bilateral manifestations^∗^	NA	NA	Hypoperfusion temporo-parieto-frontal bilateral	NA
	Left-sided manifestations^∗^	NA	NA	Hypoperfusion right brain stem, temporoparietal bilateral	NA

Marshall et al. ^∗∗^ [15]	Left sensory deficit	Weakness or paralysis	Hypoactivation right frontal lobe	Hypoactivation frontal right	NA

Spence et al. ^∗∗^ [2]	Left arm sensory deficit	Weakness or paralysis	Hypoactivation right frontal lobe	Hypoactivation frontal left	NA
	Left arm sensory deficit	Weakness or paralysis	Hypoactivation right frontal lobe	Hypoactivation frontal left	NA
	Right arm sensory deficit	Weakness or paralysis	Hypoactivation left frontal lobe	Hypoactivation frontal left	NA

NA: data nonavailable. ^∗^Limited data was available; ^∗∗^PET imaging.

## Data Availability

The data to support the findings of this study are included within the article.
